# Supplementary material for: FOXN1GFP/w Reporter hESCs Enable Identification of Integrin-β4, HLA-DR, and EpCAM as Markers of Human PSC-Derived FOXN1+ Thymic Epithelial Progenitors
Source: Stem Cell Reports. 2014 May 22;2(6):925–37. doi: 10.1016/j.stemcr.2014.04.009 (PMC4050347; doi:10.1016/j.stemcr.2014.04.009)
Supplement: Document S1. Supplemental Experimental Procedures and Figures S1–S5 [file mmc1.pdf]

Stem Cell Reports, Volume *2*

Supplemental Information

# *FOXN1*$^{GFP/w}$ Reporter hESCs Enable Identification of Integrin-β4, HLA-DR, and EpCAM as Markers of Human PSC-Derived FOXN1$^{+}$ Thymic Epithelial Progenitors

Chew-Li Soh, Antonietta Giudice, Robert A. Jenny, David A. Elliott, Tanya Hatzistavrou, Suzanne J. Micallef, Korosh Kianizad, Natalie Seach, Juan Carlos Zúñiga-Pflücker, Ann P. Chidgey, Alan Trounson, Susan K. Nilsson, David N. Haylock, Richard L. Boyd, Andrew G. Elefanty, and Edouard G. Stanley

## Supplemental Experimental Procedures

***FOXN1* targeting vector construction:** The *FOXN1* targeting vector was assembled using standard cloning techniques and Red/ET Recombination technology (Gene Bridges). DNA fragments representing the 5' and 3' homology arms were derived from bacterial artificial chromosome (BAC) RP11-915B21 spanning the human *FOXN1* genomic locus (Roswell Park Cancer Institute). *AscI* and *ClaI* restriction endonuclease sites were introduced during recombineering steps to facilitate subcloning of a *GFP-PGK-neo*$^{R}$ reporter/selection cassette into a pBR322-based targeting vector backbone, while *SwaI* restriction sites were introduced to allow linearization of the construct.

**Generation of FOXN1$^{GFP/w}$ hESC Reporter lines:** $10^{7}$ HES3 (Richards et al., 2002) or MEL1 (Millipore catalogue no. SCC020) hESCs were electroporated with 25μg of the *FOXN1* targeting vector and subjected to G418 (Geneticin, Gibco) selection. Resultant G418 resistant colonies were screened for homologous recombination events using a PCR-based strategy incorporating the forward primer *Neo4* (5'-cgatgcctgcttgccgaatatc-3') and the reverse primer *FOXN1 26063r* (5'-gcttgactttgacctctgaacc-3') (*primer pair b*, **Figures 1A and 1B**). Six (of 134) MEL1 and three (of 144) HES3 correctly targeted clones were obtained. Homologous integration of the 5' homology arm was confirmed by PCR amplification using the forward primer *FOXN1 10546f* (5'-cattcccagaccccgagtttccttg-3') and the reverse primer *GFP Reverse2* (5'-ccggtgaacagctcctcgcccttgc-3') (*primer pair a*, **Figures 1A and 1B; Figure S1A**). The loxP-flanked *neo*$^{R}$ cassette was removed by Cre recombinase-mediated excision as previously described (Davis et al., 2008). Sub-clones that had excised the *neo*$^{R}$ gene were identified using the PCR primer *GFP1* (5'-gtgctgctgcccgacaaccactac-3') in combination with the reverse primer *FOXN1 26063r*, which generated a product that spanned the site formally occupied by the *neo*$^{R}$ cassette (*primer pair c*, **Figures 1A and 1B; Figure S1A**). Correctly targeted clones that had excised the selectable marker were single cell cloned as previously described (Davis et al., 2008).

**Antibodies used in this study:** Primary antibodies used were mouse anti-human E-CADHERIN (Invitrogen), mouse anti-human SSEA-4 (Chemicon International), mouse anti-human TRA-1-60 (Chemicon International), mouse anti-human PDGFRα (BD Pharmingen), mouse anti-human CD104 (BD Pharmingen), fluorescein isothiocyanate (FITC)-conjugated mouse anti-human CD9 (BD Pharmingen), phycoerythrin (PE)-conjugated mouse anti-human EpCAM (BD Biosciences), and allophycocyanin (APC)-conjugated mouse anti-human HLA-DR (BD Pharmingen). Unconjugated primary antibodies were detected with either APC- or PE-conjugated goat anti-mouse IgG (BD Biosciences).

**Intracellular Flow Cytometry:** Following dissociation and filtration, cells were fixed and permeabilized with BD Cytofix/Cytoperm (BD Biosciences) at 4°C for 20 min. Cells were washed and stained with primary antibody or an isotype control diluted in 1X BD Perm/Wash (BD Biosciences) at 4°C for 30 min. Unconjugated OCT4 and FOXN1 primary antibodies were detected with APC-conjugated goat anti-mouse IgG (BD Pharmingen). Cells were incubated with secondary antibodies at 4°C for 30 min in the dark. Antibody staining was quantified using the BD FACSCalibur. Forward and side scatter were used to identify cells that were viable immediately prior to the initial fixation process. Flow cytometric analysis was performed using Flowlogic Software (Inivai Technologies).

**Quantitative Real-Time Polymerase Chain Reaction (q-PCR):** Total RNA was prepared using High Pure RNA Isolation Kit (Roche), according to manufacturer's instructions. First-strand cDNA was reverse-transcribed from RNA samples with random hexamer priming using SuperScript III reagents (Invitrogen), as previously described (Pick et al., 2007). Q-PCR was performed using TaqMan Gene Expression Assays with TaqMan Universal PCR Master Mix (Applied Biosystems) on the 7500 Fast Real Time PCR System (Applied Biosystems) under standard cycling parameters according to manufacturer's instructions. The TaqMan Gene Expression Assays employed in this study were *FOXN1* (Hs00186096_m1), *GAPDH* (Hs99999905_m1), *FOXA2* (Hs00232764_m1), *SOX17* (Hs00751752_s1), *HOXA3* (Hs00601076_m1), *PAX9* (Hs Hs00196354_m1), *FGFR2* (Hs01552926_m1), *INVOLUCRIN* (Hs00902520_m1), *KERATIN-10* (Hs00166289_m1), *KERATIN-5* (Hs00361185_m1), *KERATIN-8* (Hs01670053_m1) and *GCM2* (Hs00171702_m1). The comparative cycle threshold (Ct) method was used to analyze data, with gene expression levels normalized against input determined by reference to the *GAPDH* housekeeping gene, with an arbitrary multiplier of 10,000 for sample normalization, as previously published (Pick et al., 2007).

**FOXN1-GFP$^+$ TEC progenitor - CD34$^+$CD7$^+$ proT-cell co-culture assays:** Sorted CD34$^+$ cells from Lin$^-$ UCB were cultured on OP9-DL1 cells for 9-10 days in OP9 Medium containing rhFLT3L (5 ng ml$^{-1}$, Peprotech), rhIL7 (5 ng ml$^{-1}$, Peprotech) and rhSCF (30 ng ml$^{-1}$, Peprotech) (Awong et al., 2009; La Motte-Mohs et al., 2005). FACS purified CD45$^+$CD34$^+$CD7$^+$ proT-cells were either cultured alone (1 x 10$^3$ cells/well) or with FOXN1-GFP$^+$ (3.5 x 10$^3$ cells/well) or GFP$^-$ (3.5 x 10$^3$ cells/well) cells in 96-well round-bottom low attachment plates (Costar) containing APEL Medium supplemented with 20% FBS, rhFLT3L (5 ng ml$^{-1}$), rhIL7 (5 ng ml$^{-1}$) and rhSCF (30 ng ml$^{-1}$). A half-media change was performed every 3-4 days. Differentiated proT-cells were analyzed for the expression of CD34, CD1a, CD5, CD4, CD8, CD3, CD45 and CD14 cell surface markers at weekly intervals. The following antibodies (all supplied by BD Pharmingen) were employed for analyses: APC-Cy7- and APC-conjugated mouse anti-human CD45, PE-Cy7- and APC-conjugated mouse anti-human CD34, PE-conjugated mouse anti-human CD7, PE-conjugated mouse anti-human CD1a, Biotin-conjugated mouse anti-human CD5, APC-conjugated mouse anti-human CD4, APC- and PE-conjugated mouse anti-human CD8, APC-conjugated mouse anti-human CD3 and PE-conjugated mouse anti-human CD14. The biotin-conjugated CD5 was detected with a Streptavidin-PE-conjugated secondary antibody.

## References for Supplemental Material


Awong G., Herer E., Surh C.D., Dick J.E., La Motte-Mohs R.N., and Zuniga-Pflucker J.C. (2009). Characterization in vitro and engraftment potential in vivo of human progenitor T cells generated from hematopoietic stem cells. Blood *114*, 972-982.

Davis R.P., Ng E.S., Costa M., Mossman A.K., Sourris K., Elefanty A.G., and Stanley E.G. (2008). Targeting a GFP reporter gene to the MIXL1 locus of human embryonic stem cells identifies human primitive streak-like cells and enables isolation of primitive hematopoietic precursors. Blood *111*, 1876-1884.

La Motte-Mohs R.N., Herer E., and Zuniga-Pflucker J.C. (2005). Induction of T-cell development from human cord blood hematopoietic stem cells by Delta-like 1 in vitro. Blood *105*, 1431-1439.

Pick M., Azzola L., Mossman A., Stanley E.G., and Elefanty A.G. (2007). Differentiation of human embryonic stem cells in serum-free medium reveals distinct roles for bone morphogenetic protein 4, vascular endothelial growth factor, stem cell factor, and fibroblast growth factor 2 in hematopoiesis. Stem Cells *25*, 2206-2214.

Richards M., Fong C.Y., Chan W.K., Wong P.C., and Bongso A. (2002). Human feeders support prolonged undifferentiated growth of human inner cell masses and embryonic stem cells. Nat. Biotechnol. *20*, 933-936.
