# Supplementary material for: FOXN1GFP/w Reporter hESCs Enable Identification of Integrin-β4, HLA-DR, and EpCAM as Markers of Human PSC-Derived FOXN1+ Thymic Epithelial Progenitors
Source: Stem Cell Reports. 2014 May 22;2(6):925–37. doi: 10.1016/j.stemcr.2014.04.009 (PMC4050347; doi:10.1016/j.stemcr.2014.04.009)
Supplement: Document S2. Article plus Supplemental Information [file mmc4.pdf]

Stem Cell Reports
Resource

OPEN ACCESS

FOXN1$^{GFP/w}$ Reporter hESCs Enable Identification of Integrin-β4, HLA-DR, and EpCAM as Markers of Human PSC-Derived FOXN1^{+} Thymic Epithelial Progenitors

Chew-Li Soh,[1] Antonietta Giudice,[1] Robert A. Jenny,[1] David A. Elliott,[2] Tanya Hatzistavrou,[1] Suzanne J. Micallef,[1] Korosh Kianizad,[3] Natalie Seach,[1] Juan Carlos Zúñiga-Pflücker,[3] Ann P. Chidgey,[1] Alan Trounson,[4] Susan K. Nilsson,[5,6] David N. Haylock,[5,6] Richard L. Boyd,[1] Andrew G. Elefanty,[1,2] and Edouard G. Stanley[1,2,7,*]

[1]Department of Anatomy and Developmental Biology, Monash University, Clayton, VIC 3800, Australia
[2]Murdoch Childrens Research Institute, The Royal Children's Hospital, Parkville, VIC 3052, Australia
[3]Sunnybrook Research Institute, Department of Immunology, University of Toronto, Toronto, ON M4N 3M5, Canada
[4]California Institute for Regenerative Medicine, San Francisco, CA 94107, USA
[5]Materials Science and Engineering, Commonwealth Scientific and Industrial Research Organisation, Clayton, VIC 3168, Australia
[6]Australian Regenerative Medicine Institute, Monash University, Clayton, VIC 3800, Australia
[7]Department of Paediatrics, University of Melbourne, Parkville, VIC 3050, Australia
*Correspondence: ed.stanley@mcri.edu.au
http://dx.doi.org/10.1016/j.stemcr.2014.04.009
This is an open access article under the CC BY license (http://creativecommons.org/licenses/by/3.0/).

SUMMARY

Thymic epithelial cells (TECs) play a critical role in T cell maturation and tolerance induction. The generation of TECs from in vitro differentiation of human pluripotent stem cells (PSCs) provides a platform on which to study the mechanisms of this interaction and has implications for immune reconstitution. To facilitate analysis of PSC-derived TECs, we generated hESC reporter lines in which sequences encoding GFP were targeted to *FOXN1*, a gene required for TEC development. Using this *FOXN1*$^{GFP/w}$ line as a readout, we developed a reproducible protocol for generating FOXN1-GFP^{+} thymic endoderm cells. Transcriptional profiling and flow cytometry identified integrin-β4 (ITGB4, CD104) and HLA-DR as markers that could be used in combination with EpCAM to selectively purify FOXN1^{+} TEC progenitors from differentiating cultures of unmanipulated PSCs. Human FOXN1^{+} TEC progenitors generated from PSCs facilitate the study of thymus biology and are a valuable resource for future applications in regenerative medicine.

INTRODUCTION

T cells undergo most of their development in the thymus, the primary lymphoid organ that regulates their differentiation and maturation from blood-borne bone-marrow-derived precursors and appropriate selection for the induction of self-tolerance (Anderson et al., 2007). Thymic function is critically dependent on thymic epithelial cells (TECs), the most abundant cellular constituent of the stromal microenvironment. TECs are classified as two morphologically and functionally distinct subsets based on their localization to the thymic cortex (cTECs) or medulla (mTECs). TEC development and identity require the forkhead-box transcription factor *Foxn1*, which, in the mouse, demarcates the prospective thymic primordium within the third pharyngeal pouch at embryonic day 11.5 (E11.5). Loss of *Foxn1* results in a "nude" phenotype in mice and rats (Nehls et al., 1994, 1996) and in humans (Pignata et al., 1996), characterized by congenital hairlessness and defective TEC differentiation, the latter of which results in the absence of functional T cells and severe immunodeficiency.

T cell insufficiency is associated with other congenital thymic hypoplasias, such as DiGeorge syndrome (Jerome and Papaioannou, 2001), age-related thymus atrophy, or cytoablative therapy-induced thymic involution. In the last case, patients undergoing high-dose chemotherapy often experience chronic immunosuppression, predisposing them to a host of opportunistic infections. In all of these instances, replenishment of the thymic epithelial compartment might provide an avenue to augment thymus function and boost T cell output.

The derivation of tissues by in vitro differentiation of pluripotent stem cells (PSCs) has been advanced as a platform for use in the emerging fields of cell therapy and regenerative medicine. PSCs possess the capacity to give rise to the three embryonic germ layers, including the definitive endoderm, the precursor of thymic epithelium. PSC differentiation protocols that promote definitive endoderm formation (D'Amour et al., 2005; Kubo et al., 2004; Tada et al., 2005; Yasunaga et al., 2005) and the subsequent generation of posterior foregut derivatives, including pancreatic cells (D'Amour et al., 2006; Kroon et al., 2008) and hepatocytes (Cai et al., 2007; Gouon-Evans et al., 2006), are well established. Similarly, strategies designed to generate anterior foregut derivatives, such as the lung, thyroid, and thymus, have also been reported (Green et al., 2011; Lai and Jin, 2009; Longmire et al.,

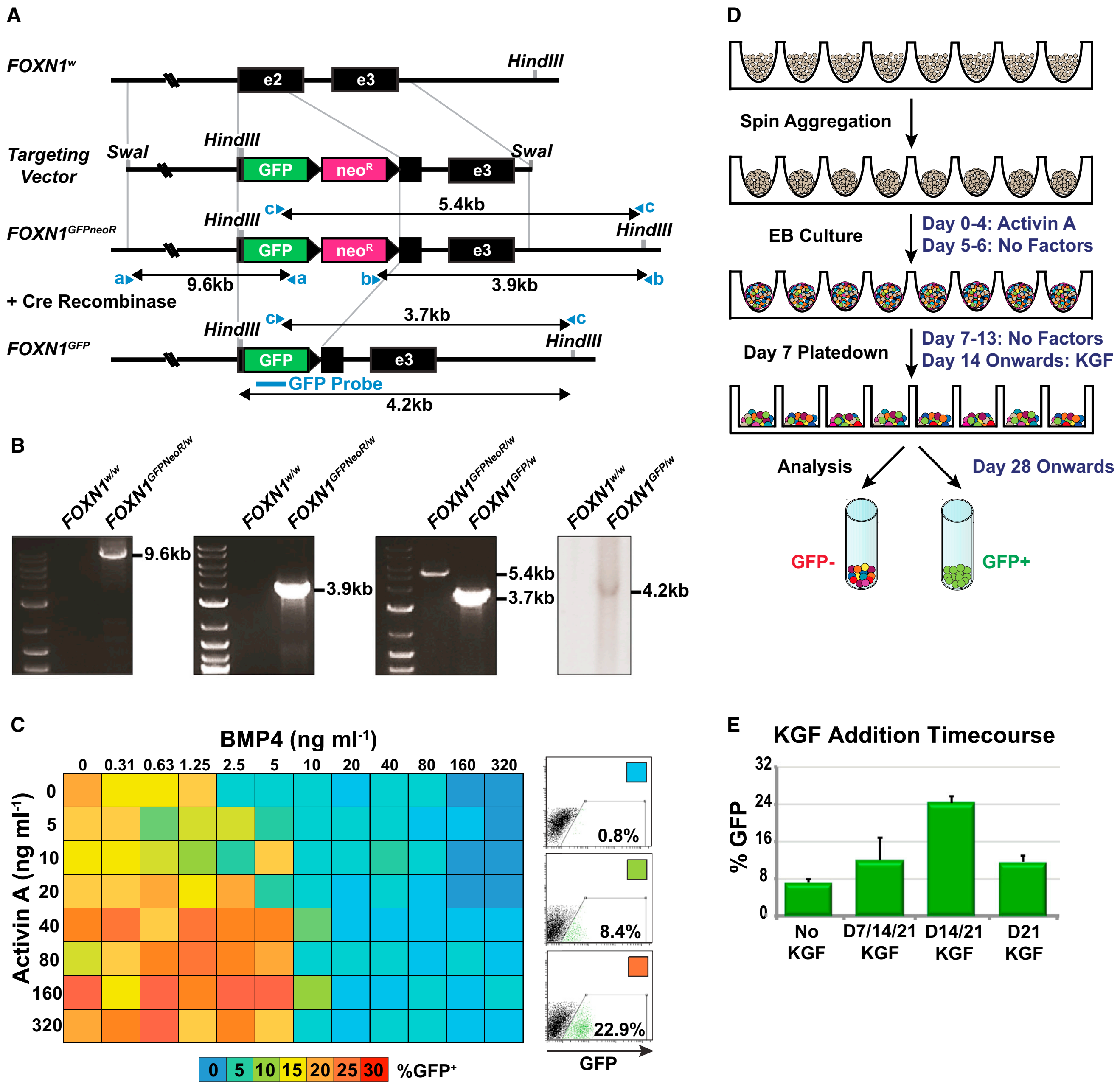

Figure 1. Targeted *FOXN1*$^{GFP/w}$ hESCs Facilitate Monitoring of Thymic Differentiation toward FOXN1-GFP$^+$ Cells

(A) Targeting strategy used to insert sequences encoding GFP into exon 2 (e2) of the *FOXN1* gene locus via homologous recombination. The selection cassette included a neomycin resistance gene (*neo*^{R}) flanked by loxP sites (black triangles). *SwaI* restriction endonuclease was used to linearize the vector prior to electroporation into hESCs. The locations of PCR primer pairs (a, b, and c) and a GFP probe (blue line) used to characterize targeted clones are also indicated.

(B) PCR analyses of HES3-derived *FOXN1*$^{GFP/w}$ hESCs with primer pairs (a and b) spanning the junctions between the targeting vector and genomic DNA generated bands of 9.6 kb (5′ end) and 3.9 kb (3′ end) from targeted *FOXN1*$^{GFPneoR/w}$, but not from wild-type hESCs. PCR analysis with primer pair c flanking the *neo*^{R} gene generated bands of 3.7 kb compared to 5.4 kb, indicating excision of the *neo*^{R} cassette. Southern blot analysis of HindIII-digested *FOXN1*$^{GFP/w}$ genomic DNA using the GFP probe detected a single 4.2 kb fragment, demonstrating integration of a single copy of the *FOXN1*-targeting vector.

(C) Heatmap summarizing the results of flow cytometry experiments assessing the percentage of GFP$^+$ cells from days 30–35 embryoid bodies generated with the indicated BMP4 and Activin A concentrations. Data were derived from three independent experiments. FACS profiles representing selected conditions are shown on the right.

(legend continued on next page)

2012). Recently, two groups reported methods for the generation of thymic endoderm from human pluripotent stem cells (Parent et al., 2013; Sun et al., 2013). Importantly, these studies showed that differentiated mixed cultures containing thymic progenitors could mature in vivo to form grafts capable of supporting T cell development in nude (*Foxn1*$^{-/-}$) or other immunocompromised mice. However, because of the lack of appropriate surface markers, it was not possible to dissect the contributions or requirements of the various cell types present in the differentiation cultures at the time of transplantation. This deficit could be partly remedied by the availability of *FOXN1* reporter lines or surface markers that allow further fractionation of cultures containing FOXN1$^+$ cells.

We generated *FOXN1*$^{GFP/w}$ human embryonic stem cell (hESC) reporter lines that were used to develop a robust serum-free protocol for the generation of FOXN1$^+$ thymic endodermal progenitors. We found that high levels of Activin A and KGF efficiently induced the differentiation of FOXN1$^+$ cells and that these cells expressed genes involved in endoderm and thymus development. Transcriptional profiling of purified FOXN1-GFP$^+$ cells allowed the identification of several combinations of cell-surface markers that could selectively isolate FOXN1$^+$ TEC progenitor populations derived from unmodified PSC lines. Collectively, these reagents and findings represent a valuable resource for the further investigation of thymic development from pluripotent stem cells.

RESULTS

FOXN1$^{GFP/w}$ hESCs Facilitate Analysis of Thymic Differentiation In Vitro

To facilitate analysis of thymic differentiation of PSCs, we used homologous recombination to target a *GFP* reporter gene to the endogenous *FOXN1* locus in MEL1 (Millipore) and HES3 (Richards et al., 2002) hESCs (Figures 1A and 1B; Figure S1A available online). Targeted *FOXN1*$^{GFP/w}$ hESCs retained expression of pluripotent stem cell markers, formed multilineage teratomas when transplanted into immunodeficient NOD/SCID mice, and maintained normal karyotypes (Figures S1B–S1D). In order to identify conditions that favored the formation of FOXN1$^+$ endodermal cells, we differentiated *FOXN1*$^{GFP/w}$ hESCs in serum-free media as spin embryoid bodies (EBs) (Ng et al., 2008) and cross-titrated bone morphogenetic protein-4 (BMP4) and Activin A, growth factors that activate signaling pathways required for the generation and patterning of mesendoderm and have been previously shown to promote PSC differentiation in vitro (D'Amour et al., 2005; Davis et al., 2008; Kubo et al., 2004; Tada et al., 2005; Yasunaga et al., 2005). This heatmap analysis employed an iterative process (Elliott et al., 2011) and identified combinations of BMP4 and Activin A that promoted the appearance of FOXN1-GFP$^+$ cells, which also costained with the endodermal marker EpCAM (Figure 1C; Figures S2A–S2C). Based on these results and findings from experiments using different batches of Activin A (data not shown), for all subsequent experiments, we differentiated hESCs for 5 days in serum-free medium supplemented with 150 ng ml^{-1} Activin A alone (Figure 1D). These high Activin conditions were similar to those used in previously published differentiation protocols where definitive endoderm derivatives were the desired outcome (D'Amour et al., 2006; Green et al., 2011). Flow cytometric analysis of differentiating *MIXL1*$^{GFP/w}$ hESCs, which can be used to monitor mesendoderm induction (Davis et al., 2008), indicated that at differentiation day 4, over 99% of cells were MIXL1-GFP$^+$, arguing against the possibility that FOXN1-GFP$^+$ cells represented ectodermally derived keratinocytes (Figure S3A). Time course quantitative real-time PCR analysis of differentiation cultures demonstrated that from day 12, cells progressively upregulated expression of the pharyngeal pouch markers *HOXA3* and *PAX9*, as well as the thymic markers *INVOLUCRIN*, *FGFR2* (the receptor for KGF), *KERATIN-10*, and *FOXN1* itself. Expression of the general endodermal markers *FOXA2* and *SOX17* declined from a peak at day 12, consistent with expansion of a specialized anterior endoderm population that does not express these markers at high levels (Figure S3B).

Keratinocyte growth factor (KGF or FGF7), a factor previously implicated in thymic development, was required to expand the FOXN1-GFP$^+$ population once it emerged in culture, with the timing of the KGF supplementation affecting the percentage of GFP$^+$ cells obtained (Figures 1D and 1E). KGF is expressed in the thymus by the surrounding mesenchyme and by different thymocyte subsets (Rossi et al., 2007) and exerts a paracrine effect by binding to the FgfR2IIIb receptor, which, within the thymus, is expressed exclusively by TECs (Erickson et al., 2002). Moreover, exogenous administration of KGF has been shown to increase thymic cellularity by enhancing the proliferation and function of TECs (Erickson et al., 2002; Min et al., 2007; Rossi et al., 2007). After the addition of KGF, GFP$^+$

(D) Spin embryoid body protocol for generating FOXN1-GFP$^+$ cells.
(E) Summary of flow cytometric data indicating the effects of KGF addition. Following EB culture with Activin A, KGF was either added at days 7, 14, and 21, days 14 and 21, or day 21. Differentiations were performed in APEL or BPEL medium with or without 50 ng ml^{-1} noggin at days 5 and 6. Error bars show SEM for three independent experiments.

cells could be visualized using fluorescence microscopy from approximately day 25 of differentiation. Flow cytometric analysis of cells differentiated as outlined in Figure 1D indicated that the first FOXN1-GFP$^+$ cells emerged from a PDGFRα$^-$ and EpCAM$^+$ population as early as differentiation day 15 and increased in frequency thereafter (up to 38% GFP$^+$ at day 35) (Figure 2A). MEL1-derived *FOXN1*$^{GFP/w}$ hESCs differentiated with similar kinetics to the HES3-derived line, suggesting our protocol is applicable to other PSC lines (Figure 2A). Quantitative real-time PCR analysis of cells purified on the basis of GFP and EpCAM expression showed that *FOXN1* transcripts were confined to the GFP$^+$ population (Figure 2B), confirming the fidelity of the *GFP* reporter gene. This conclusion was supported by intracellular flow cytometric analysis of FOXN1 protein expression in purified GFP$^+$ and GFP$^-$ cells (Figure S3C). Prolonged culture of aggregates of fluorescence-activated cell sorting (FACS)-purified GFP$^+$EpCAM$^+$ cells indicated that GFP expression was retained for up to 3 weeks (Figure 2B).

Gene Expression Analysis of hESC-Derived FOXN1$^+$ TEC Progenitors

We next performed microarray analysis to survey genes expressed in the GFP$^+$ fraction and to search for markers that might identify FOXN1$^+$ cells in differentiating cultures derived from unmanipulated human PSCs. We first compared the global gene expression within the GFP$^+$ and GFP$^-$ EpCAM$^+$ epithelial subpopulations. This analysis identified 115 genes that were upregulated in the GFP$^+$ EpCAM$^+$ fraction compared to the GFP$^-$EpCAM$^+$ fraction (Figure 3A; see Tables S1 and S2 for the full list of genes differentially upregulated by 3-fold), and many of these have been previously associated with thymus development and/or *FOXN1* expression. These included the canonical *WNT*-signaling molecules *WNT4* and *WNT3* and the *WNT* regulator *KRM2*, the *NOTCH* ligands *DLL1*, thought to be required for crosstalk in driving T-lymphopoiesis, and *JAG2* (Candi et al., 2007), a downstream effector of the *ΔNP63* isoform of the *TP63* transcription factor, required for the proliferation of TECs and other stratified epithelia, human TEC type I cytokeratins *KRT14*, *KRT15*, and *KRT16*, and the chemokine *CXCL14* whose transcript is expressed in fetal TECs and may aid in the recruitment of T cell precursors (Liu et al., 2005). Conversely, 213 transcripts were preferentially upregulated in the GFP$^-$ EpCAM$^+$ population (Figure 3A). These included *HAND1* and *ISL1*, genes expressed during cardiac morphogenesis and in neural crest derivatives, although *ISL1* is also expressed in pancreatic endoderm, other definitive endodermal markers, such as *APOA1*, *SOX9*, and *TGFB1*, and human TEC type II cytokeratins *KRT6*, *KRT7*, and *KRT8*. Interestingly, the chemokine *CXCL12* was also upregulated within the GFP$^-$EpCAM$^+$ population. *CXCL12* is expressed within TECs and bone marrow niches, where it acts as a chemoattractant for T cell precursors, monocytes, and hematopoietic stem cells (HSCs) (Greenbaum et al., 2013; Liu et al., 2005). Overall, the GFP$^-$EpCAM$^+$ population expressed genes associated with gene ontology (GO) categories including “blood circulation” (p value < 0.002), “circulatory system process” (p value < 0.002), “cell migration” (p value < 0.003), and “cell motility” (p value < 0.006) (Figure S4A). In contrast, GFP$^+$EpCAM$^+$ cells were enriched for genes associated with *FOXN1* expression, thymus development, or epithelial-related GO categories (Figure S4A). Although it is not possible to infer from gene expression data which genes are direct targets of FOXN1, the ability to generate and purify a large number of FOXN1$^+$ cells provides an opportunity to examine this issue using techniques such as chromatin immunoprecipitation (ChIP). Last, we also compared gene expression of the GFP$^+$ EpCAM$^+$ and GFP$^-$EpCAM$^-$ populations (Figure S4B). Included among the transcripts preferentially expressed within GFP$^+$EpCAM$^+$ cells were genes with a role in thymus development (*KRM2*, *JAG2*, *WNT4*, *BCL11B*, *EFNA1*, and *EYA2*), epithelial morphogenesis (*KRT5*, *TP63*, *EpCAM*, *KRT14*, *ECAD*, *ITGB4*, and *EGFR*), and endoderm specification (*ALB* and *AFP*).

Keratin-5 and Keratin-8 have been detected within the human thymus as markers of the medulla and cortex, respectively (Laster et al., 1986; Shezen et al., 1995). Our microarray analysis demonstrated high coexpression of *Keratin-5* and its heterodimerization partner *Keratin-14* within FOXN1$^+$ (GFP$^+$EpCAM$^+$) cells (Figure 3B). The former result was confirmed by quantitative real-time PCR (Figure S4C). Interestingly, although also detected in GFP$^+$EpCAM$^+$ cells, *Keratin-8* is preferentially expressed by the GFP$^-$EpCAM$^+$ subpopulation (Figure 3A; Figure S4C). These results might suggest that the FOXN1$^+$ cells generated in our protocol more closely reflect a medullary phenotype. Finally, FOXN1$^+$ cells were devoid of genes that mark the parathyroid gland, including *GCM2*, *PTH*, and *CaSR* (Figures S4D and S4E), consistent with the observation that these two developmentally related tissues can be delineated on the basis of *FOXN1* and *GCM2* expression (Gordon et al., 2001). Similarly, other genes associated with the demarcation of thymic versus parathyroid primordia (*BMP4*, *BMPR1A*, *BMPR2*, and *NOGGIN*) were not restricted to specific subpopulations (Figure S4E).

Examination of microarray data indicated that a number of transcription factors, signaling molecules, and structural proteins implicated in TEC differentiation were preferentially expressed in GFP$^+$EpCAM$^+$ cells (Figure 3B). We were particularly interested in identifying transmembrane proteins for which well-characterized cell-surface antibodies were readily available. Such antibodies could then

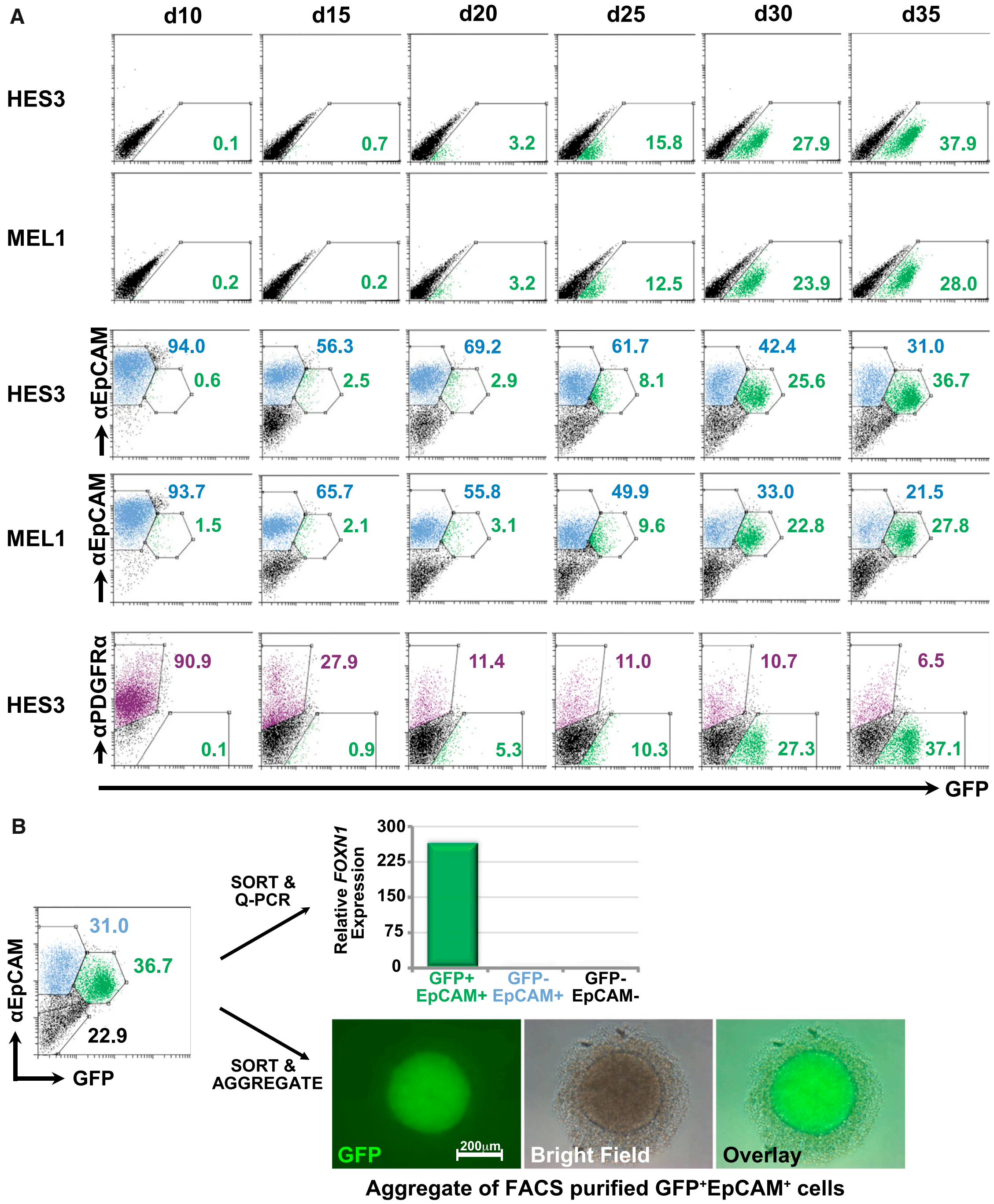

Figure 2. Activin A and KGF Induce GFP Expression in Differentiating *FOXN1*$^{GFP/w}$ hESCs

(A) Flow cytometric analysis of differentiating HES3-derived *FOXN1*$^{GFP/w}$ hESCs shows FOXN1-GFP^{+} cells emerge at day 15 and subsequently increase thereafter. FOXN1-GFP^{+} cells coexpress the thymic epithelial marker EpCAM, but not the mesodermal marker PDGFRα. Flow cytometric analysis of a second independent MEL1-derived *FOXN1*$^{GFP/w}$ hESC line demonstrated that the time course of GFP and EpCAM induction was comparable between HES3- and MEL1-derived lines.

(B) Quantitative real-time PCR analysis showed that *FOXN1* expression was confined to the GFP^{+}EPCAM^{+} fraction, with expression absent from the GFP^{-}EpCAM^{+} and GFP^{-}EpCAM^{-} subpopulations. Aggregated GFP^{+}EpCAM^{+} cells retained GFP expression for up to 3 weeks.

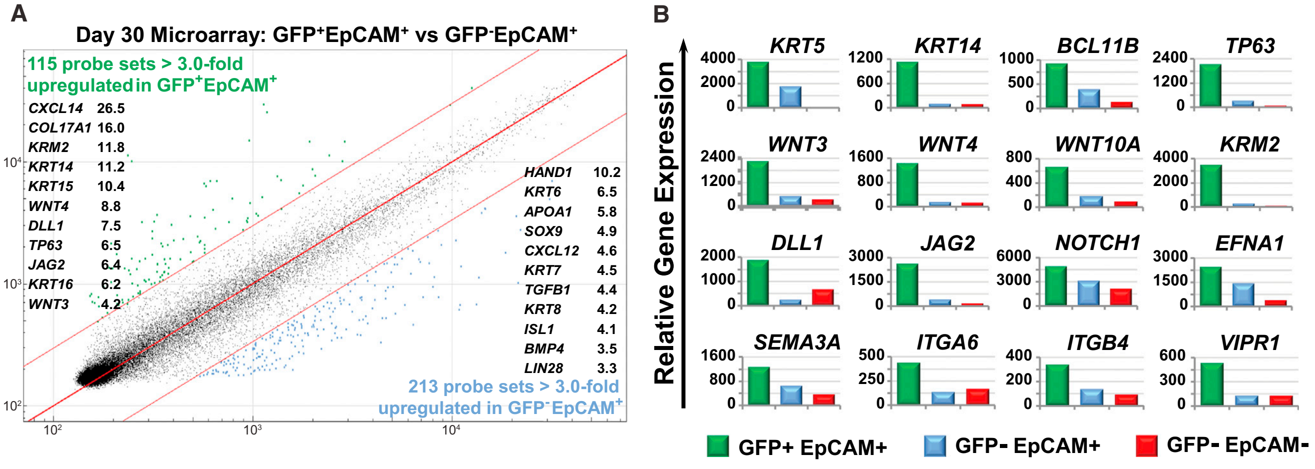

Figure 3. Microarray Analysis of FOXN1-GFP$^+$ Thymic Epithelial Progenitor Cells

(A) Comparison of gene expression in day 30 GFP$^+$EpCAM$^+$ and GFP$^-$EpCAM$^+$ cells. Selected differentially regulated key genes are listed along with the fold change in gene expression between the two populations.

(B) Genes associated with endoderm commitment and thymus development are preferentially expressed in GFP$^+$EpCAM$^+$ cells compared to GFP$^-$EpCAM$^+$ and GFP$^-$EpCAM$^-$ cells. The cell-surface marker ITGB4 was among the transcripts upregulated.

be employed to isolate FOXN1$^+$ cells from differentiating cultures of genetically unmodified PSC lines. One such gene upregulated in GFP$^+$EpCAM$^+$ cells was *integrin-β4* (*ITGB4* or *CD104*) (Figure 3B). The ITGB4 subunit commonly associates with the integrin-α6 (ITGA6) subunit to form an integrin α6β4 heterodimer that functions as a receptor for laminins within the extracellular matrix (Giancotti, 1996). *ITGB4* is expressed in normal human TECs, where it is thought to activate intracellular signaling pathways that regulate production of the thymic cytokine *IL-6*, which in turn affects TEC differentiation and thymocyte homing (Mainiero et al., 2003). Moreover, *ITGB4* is also expressed by cultured human TEC lines (Beaudette-Zlatanova et al., 2011). Time course analysis showed low expression of ITGB4 in early differentiating embryoid bodies, which preceded the appearance of FOXN1-GFP$^+$ cells, and was associated with a population that concomitantly upregulated EpCAM expression (Figure 4A). Indeed as differentiation proceeded, by day 15 the majority of EpCAM$^+$ cells coexpressed ITGB4, consistent with the expression of these markers on a broad range of epithelial tissues (Figure 4A). EpCAM$^+$, FOXN1-GFP$^+$ cells appearing from differentiation day 20 onward expressed high levels of ITGB4 (ITGB4^{hi}). Furthermore, as the number of FOXN1-GFP$^+$ cells increased in response to KGF treatment, GFP expression became more clearly associated with the ITGB4^{hi}EpCAM$^+$ subpopulation. Quantitative real-time PCR analysis of FACS-purified day 34 cells derived from the differentiation of *FOXN1$^{GFP/w}$* hESCs confirmed that *FOXN1* gene expression was largely confined to the ITGB4^{hi}EpCAM$^+$ population and that expression levels were comparable to those seen in cells sorted on the basis of GFP expression alone (Figure 4B). Negligible *FOXN1* expression was observed in sorted populations that expressed intermediate or low levels of *ITGB4* or were EpCAM$^-$.

To explore the utility of these cell-surface makers for monitoring developing TECs, we examined other human pluripotent stem cell lines, including MEL1-derived *FOXN1$^{GFP/w}$* hESCs, genetically unmodified H9 hESCs (Thomson et al., 1998) and DF19-9-7T human iPSCs (Yu et al., 2009) (Figure 4B). All human PSC lines generated ITGB4^{hi}EpCAM$^+$ cells with parallel kinetics, although the proportion of this population varied between different cell lines. Cell populations that expressed ITGB4 and EpCAM but were not ITGB4^{hi} (designated ITGB4$^+$EpCAM$^+$) varied in composition between different PSC lines. Nevertheless, we found *FOXN1* expression to be solely confined to those EpCAM$^+$ cells that were ITGB4^{hi} (Figures 4B and 4C). Overall, flow cytometric analysis of HES3 and MEL1 *FOXN1$^{GFP/w}$* cultures indicated that FOXN1-GFP$^+$ cells comprised over 80% of the ITGB4^{hi}EpCAM$^+$ fraction (Figure S4F). Conversely, 95% of the GFP$^+$ population was found to be ITGB4^{hi}EpCAM$^+$, testifying to the close association between these markers and *FOXN1* expression (Figure S4G).

In addition to those markers identified by transcriptional profiling, we examined the expression of the TEC-associated receptor, *HLA-DR*: a human major histocompatibility complex class II (MHCII) cell-surface receptor encoded by the human leukocyte antigen (HLA) complex (Walsh et al., 2003). Within the thymus, the primary function of

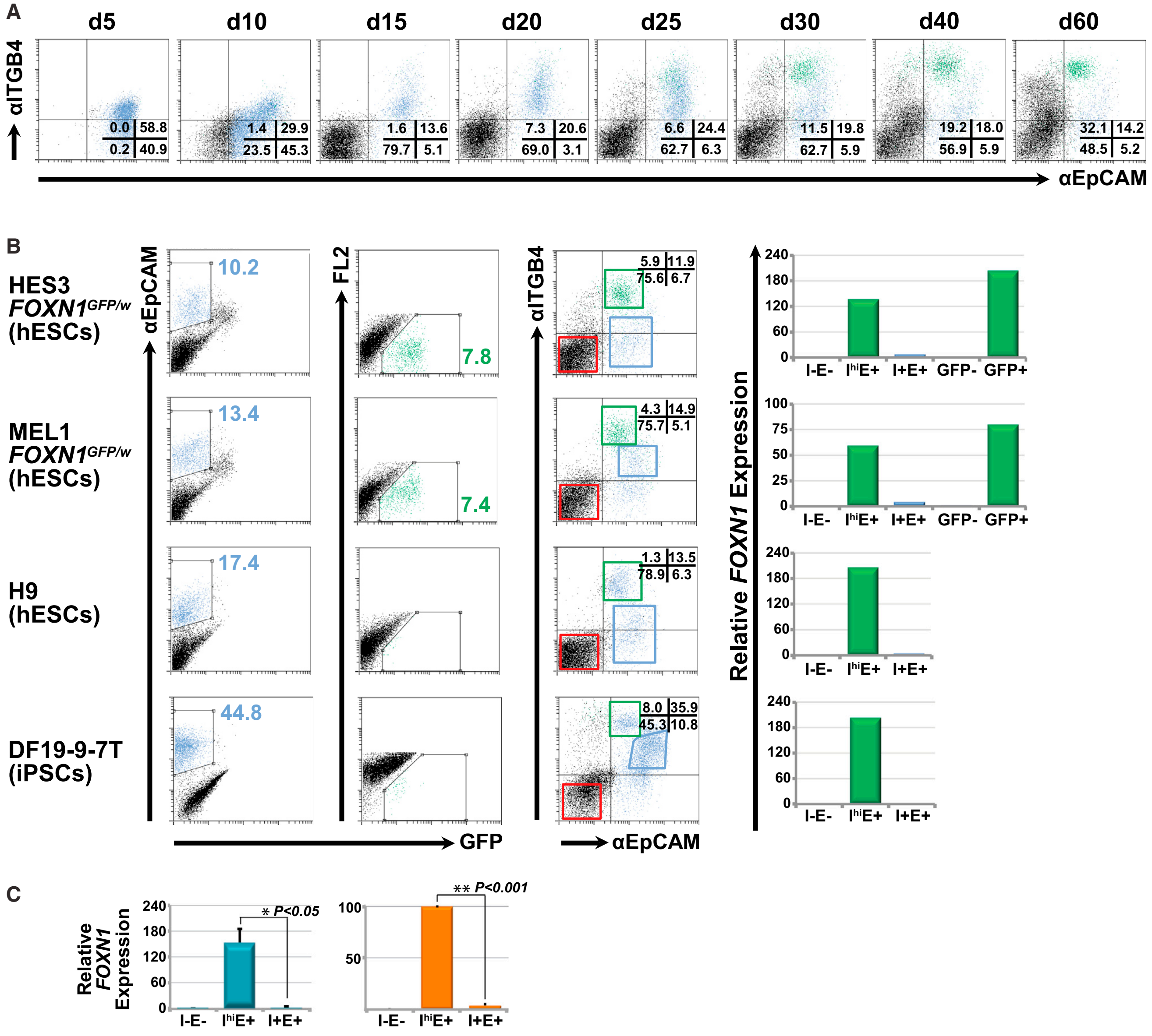

Figure 4. Integrin-β4 and EpCAM Identify FOXN1⁺ Thymic Epithelial Progenitor Cells

(A) Time course analysis of differentiating HES3-derived *FOXN1*$^{GFP/w}$ cells demonstrated that FOXN1-GFP^{+} cells could be identified on the basis of integrin-β4 (ITGB4) and EpCAM expression. GFP expression was localized to EpCAM^{+} cells that expressed high levels of ITGB4. EpCAM^{+} cells are shown gated in light blue, whereas GFP^{+} cells are shown gated in green. Numbers indicate the percentage of cells in the corresponding quadrants.

(B) Quantitative real-time PCR analysis confirmed that *FOXN1* transcripts were restricted to the ITGB4^{hi}EpCAM^{+} population differentiated from HES3-derived *FOXN1*$^{GFP/w}$ cells and were present at levels similar to those observed in cells isolated on the basis of FOXN1-GFP expression alone. *FOXN1* expression in differentiating MEL1-derived *FOXN1*$^{GFP/w}$ cells and H9 hESCs or DF19-9-7T human iPSCs was also confined to the ITGB4^{hi}EpCAM^{+} population. Colored boxes represent the gating strategy used to isolate subpopulations for flow cytometric sorting. I, ITGB4; E, EpCAM. −, +, and hi indicate negative, positive, and high levels of expression, respectively.

(C) Histograms demonstrating the summary of raw (left panel) and normalized (right panel) data from individual quantitative real-time PCR experiments shown in Figure 4B (n = 4). Data are shown as the mean ± SEM. p values were calculated using a two-tailed Student's t test.

HLA-DR, similar to other *MHCII* genes, is to present peptide antigens during positive and negative T cell selection in the thymic cortex and medulla (Yang et al., 2006). TECs are unique in that they constitutively express *MHCII* without the need for interferon-γ (IFN-γ) stimulation (Drukker et al., 2002; Yang et al., 2006), consistent with the fact

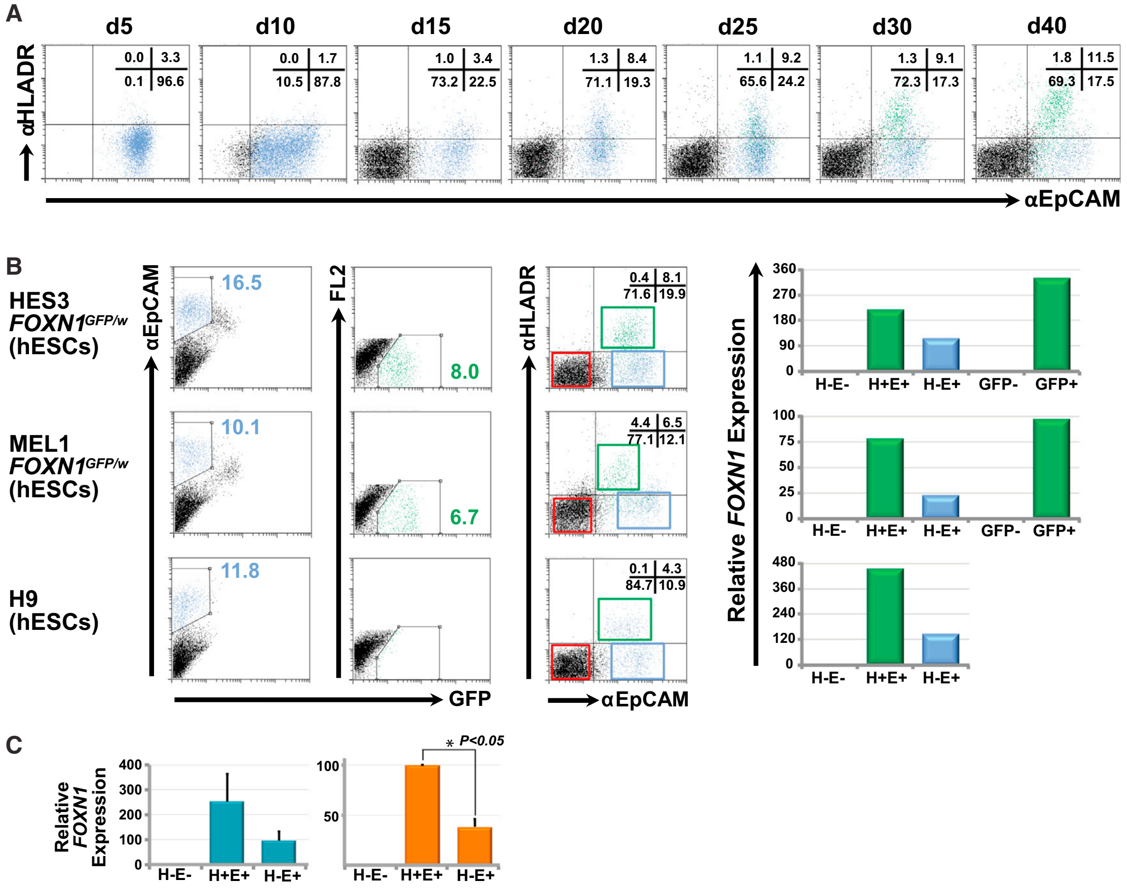

Figure 5. HLA-DR and EpCAM Identify PSC-Derived FOXN1$^+$ Thymic Epithelial Progenitor Cells

(A) Time course analysis of differentiating HES3-derived *FOXN1$^{GFP/w}$* cells demonstrated that FOXN1-GFP$^+$ cells could be identified on the basis of coexpression of HLA-DR and EpCAM. EpCAM$^+$ cells are shown gated in light blue, whereas GFP$^+$ cells are shown gated in green. Numbers indicate the percentage of cells in the corresponding quadrants.

(B) Quantitative real-time PCR analysis confirmed that *FOXN1* transcripts were substantially enriched in the HLA-DR$^+$EpCAM$^+$ population differentiated from HES3-derived *FOXN1$^{GFP/w}$* cells and were present at levels similar to those observed in FOXN1-GFP$^+$ cells. *FOXN1* expression in differentiating MEL1-derived *FOXN1$^{GFP/w}$* cells and H9 hESCs was also enriched in the HLA-DR$^+$EpCAM$^+$ population. Colored boxes represent the gating strategy used to isolate subpopulations for flow cytometric sorting. H, HLA-DR; E, EpCAM.

(C) Histograms demonstrating the summary of raw (left panel) and normalized (right panel) data from individual quantitative real-time PCR experiments shown in Figure 5B (n = 3). Data are shown as the mean ± SEM. p values were calculated using a two-tailed Student's t test.

that IFN-γ-deficient mice undergo normal thymic development (Dalton et al., 1993). To explore whether HLA-DR could also identify FOXN1-GFP$^+$ cells, we performed flow cytometry to monitor the expression of HLA-DR and EpCAM over a 40 day differentiation time course (Figure 5A). Similar to previous results (Drukker et al., 2002), HLA-DR was not expressed on the surface of undifferentiated hESCs (data not shown) or during the early stages of embryoid body formation. Expression was first observed from approximately day 15 and increased thereafter. By day 40, we detected a distinct population of HLA-DR$^+$ EpCAM$^+$ cells that contained the majority of FOXN1-GFP$^+$ cells (Figure 5A). Furthermore, all HLA-DR$^+$ cells were also EpCAM$^+$, suggesting that HLA-DR is restricted to only thymic epithelial cells in our culture system. Consistent with past studies, HLA-DR expression on FOXN1-GFP$^+$ TEC progenitors was not upregulated in response to IFN-γ stimulation. Conversely, as expected, IFN-γ treatment induced expression of HLA-DR in the non-GFP population (Figure S4H). Finally, quantitative real-time PCR analysis was performed on day 30 differentiated cells purified on the basis of HLA-DR and EpCAM expression (Figure 5B). This analysis showed that *FOXN1* transcripts were greatly enriched in the HLA-DR$^+$EpCAM$^+$

fraction, with expression levels comparable to those seen in cells sorted on the basis of GFP alone. The veracity of this result was confirmed in similar analyses of HLA-DR$^+$ EpCAM$^+$ cells isolated from differentiating cultures of MEL1 *FOXN1*$^{GFP/w}$ and H9 hESC lines (Figures 5B and 5C). Similar to results obtained for cells isolated on the basis of ITGB4 and EpCAM expression, flow cytometric analysis indicated FOXN1-GFP$^+$ cells comprised over 70% of cells within the HLA-DR$^+$EpCAM$^+$ fraction (Figure S4I). The clear association of *FOXN1* expression with HLA-DR further substantiates the thymic identity of cells generated using our protocol.

The upregulation of HLA-DR in conjunction with gene expression analyses indicating the presence of *NOTCH* signaling receptors and ligands prompted us to test whether FOXN1-GFP$^+$ cells generated with our differentiation protocol might be competent to promote the development of T lineage cells from human CD34$^+$ hematopoietic progenitors. To test this hypothesis, we cocultivated purified FOXN1-GFP$^+$ with human umbilical cord blood-derived CD34$^+$CD7$^+$ T lineage progenitors, a cell type previously shown to differentiate into CD4$^+$ or CD8$^+$ T cells (Awong et al., 2009; La Motte-Mohs et al., 2005). Over a 4 week coculture period, we analyzed the hematopoietic component for expression of CD1a, CD5, CD4, CD8, and CD3, markers of immature T lineage progenitors, differentiating proT-cells and mature T cells. Although we observed the induction of CD1a, CD5, CD4, and CD8 expression, the differentiation step was not specifically dependent on the presence of FOXN1-GFP$^+$ cells (Figures S5A–S5C and S5E). Moreover, we did not observe convincing expression of the T-lineage-specific marker CD3 (Figure S5D). Rather, a significant proportion of CD34$^+$ cells differentiated into cells with a myeloid phenotype (CD45$^+$CD14$^+$) (Figure S5E). Hence, although our human PSC-derived cells possess a TEC-like phenotype, we believe that they represent a stage that is too immature to drive T cell differentiation. Further investigations into the growth factors necessary for the commitment of FOXN1$^+$ TEC progenitors into mature TECs in our culture system are therefore required.

DISCUSSION

We devised a simple method for generating and isolating FOXN1$^+$ thymic epithelial progenitor cells differentiated from human pluripotent stem cells. We inserted a GFP reporter gene into the locus encoding the key thymic transcription factor *FOXN1*, to facilitate monitoring of the early steps involved in human thymus specification. This reporter gene faithfully identified cells expressing *FOXN1* and enabled isolation of FOXN1$^+$ thymic progenitors from heterogeneous differentiation cultures. Although targeted lines represent an extremely useful research tool, genetically manipulated lines are undesirable for potential future clinical use. Moreover, the technical requirements for genetic modification make the application of this approach impractical for the ever-expanding number of available pluripotent stem cell lines. With these points in mind, we interrogated the expression profile of FACS-purified FOXN1-GFP$^+$ cells through microarray analysis with the aim of identifying genes that could serve as a surrogate marker of *FOXN1* expression. From this screen, we identified ITGB4 as a cell-surface marker that, in conjunction with EpCAM, could be used to isolate FOXN1$^+$ cells from genetically unmodified hESCs and from human iPSCs. Similarly, our analysis showed that HLA-DR and EpCAM were also specific markers of FOXN1$^+$ TEC progenitors. Taken together, our study provides a model system to investigate the signaling pathways required for TEC commitment and differentiation.

Gene expression analysis revealed that cells differentiated for up to 30 days expressed markers implicated in endoderm specification and TEC development but lacked the expression of important late-stage TEC markers associated with functional maturation. In particular, relative to human pediatric thymic stroma (data not shown), in vitro-derived FOXN1$^+$ TEC progenitors showed low expression of *autoimmune regulator* (*AIRE*), a gene critical for intrathymic expression of tissue-restricted antigens, which in turn is required to induce tolerance to peripheral antigens (Anderson et al., 2007). In addition, CD80, a costimulatory molecule expressed on functionally mature mTECs (Derbinski et al., 2005), was also not enriched on FOXN1$^+$ cells. These data indicated that the thymic endoderm produced using our culture conditions most likely represented an early stage of ontogeny in which overt thymic functional characteristics were yet to be acquired.

The latter stages of thymic epithelial development are dependent on the presence of hematopoietic cells that, in the mouse, infiltrate the thymic primordium at embryonic day 12, soon after the onset of *FOXN1* expression. Subsequent to this, the thymic endoderm and lymphoid progenitors engage in signaling crosstalk that ultimately yields functionally mature TECs and educated T cells. The Notch-Delta system is a central signaling mechanism implicated in directing this crosstalk (Mohtashami et al., 2010). In the thymus, *Notch1* expressed on hematopoietic progenitors interacts with *Dll1* or *Dll4* on TECs to either maintain or induce T-lineage commitment and differentiation. Because of this, we tested whether coculture of FOXN1$^+$ thymic endoderm with hematopoietic progenitor cells could provide an environment whereby both cell types could undergo further maturation. However, under the conditions used, we saw no evidence of ongoing

T cell differentiation. Moreover, analysis of these cocultures suggested that the viability of thymic endoderm cells was compromised, suggesting that further work will be required to define conditions that are permissive for the continued growth and development of both hematopoietic and endodermal cells types.

Recently, Parent et al. (2013) and Sun et al. (2013) reported methods for the generation of thymic endoderm from pluripotent stem cells. Following induction of definitive endoderm by Activin A treatment, both groups could promote the appearance of anterior foregut and subsequently pharyngeal endoderm by carefully choreographed manipulation of WNT, retinoic acid, and BMP4 signaling, mirroring the inductive and repressive actions of these pathways during early thymic ontogeny. Both studies showed that mixed differentiation cultures containing thymic progenitors could instruct T cell development in xenotransplantation models. In our differentiation protocol, the activities of the WNT, retinoic acid, and BMP4 pathways were not deliberately manipulated with exogenous growth factors or inhibitors, potentially explaining the inability of our FOXN1^{+} cells to upregulate markers of late-stage TEC differentiation or to support T cell development. Having said this, we did not formally test whether heterogeneous non-FACS-purified populations containing FOXN1^{+} cells could differentiate further following transplantation or indeed support T cell development in vitro. Consequently, the potential functionality and differentiation status of the cells generated using our simple method is difficult to directly compare with cells described by Parent et al. (2013) and Sun et al. (2013), where mixed cultures were used for in vivo studies.

The cell lines and surface markers reported in this study provide a facile platform on which to begin the process of identifying key events and critical cell populations required to derive fully functional TECs from PSCs. These *FOXN1*$^{GFP/w}$ reporter lines represent a unique research tool to analyze human thymus development, whereas the cell-surface marker combinations recognized by antibodies against ITGB4, HLA-DR, and EpCAM can be used to purify TEC progenitors from cultures of differentiating unmanipulated PSCs. Although the exogenous growth factors required for ongoing differentiation of FOXN1^{+} progenitor cells into functionally mature TECs remain to be established, our study contributes critical tools for efforts to generate this clinically important cell type.

EXPERIMENTAL PROCEDURES

Construction of the *FOXN1*-Targeting Vector

The *FOXN1*-targeting vector was assembled using standard cloning techniques and *Gene Bridges* Red/ET Recombination technology (see the Supplemental Experimental Procedures). The final 18.2 kb *FOXN1*-targeting vector comprised a 9.6 kb 5′ homology arm, a *GFP*-coding sequence, a loxP-flanked neomycin resistance gene driven by the mouse phosphoglycerate kinase (*PGK*) gene promoter, a 3.9 kb 3′ homology arm, and a pBR322-based plasmid backbone. The targeting vector was linearized by *SwaI* digestion prior to electroporation.

Generation, Identification, and Characterization of Targeted *FOXN1*$^{GFP/w}$ hESCs

The *FOXN1*-targeting vector was electroporated into hESCs as previously described (Costa et al., 2007) and as detailed in the Supplemental Experimental Procedures. The loxP-flanked *neo*^{R} cassette was removed by Cre recombinase-mediated excision as previously described (Davis et al., 2008). For one of the two independently derived targeted hESC lines (HES3 *FOXN1*$^{GFP/w}$ and MEL1 *FOXN1*$^{GFP/w}$), Southern blot analysis with a labeled GFP probe (Figure 1A) was used to confirm the presence of a single-integration event (Figure 1B). Karyotype analysis was performed by the Cytogenetics Department at Southern Cross Pathology, Monash Medical Centre. Teratoma formation and analysis were performed as previously described (Costa et al., 2005). Animal experiments were conducted under the approval of the Monash University Animal Ethics Committee (number SOBSA/MIS/2009/07).

hESC and iPSC Culture and Differentiation

hESC lines (HES3, MEL1, and H9 [Thomson et al., 1998]) and the induced pluripotent stem cell (iPSC) line DF19-9-7T (Yu et al., 2009) were maintained as previously described (Costa et al., 2008). hESCs and iPSCs were differentiated as spin embryoid bodies in serum-free media as previously described (Ng et al., 2008). Briefly, 1 day prior to differentiation, cells were passaged onto a new tissue culture flask seeded with low density (1×10^{4} cells/cm^{2}) mouse embryonic fibroblasts. At day 0, cells were harvested and deposited into each well (3×10^{3} cells/well) of a 96-well round-bottom nonadherent plate (Nunc) and briefly centrifuged to promote cell aggregation.

Heatmap experiments were performed using APEL medium (Ng et al., 2008), supplemented with 0–320 ng ml^{-1} Activin A (R&D Systems) and 0–320 ng ml^{-1} bone morphogenetic protein-4 (BMP4; R&D Systems) in 96-well round-bottom low-attachment plates (Costar) (Elliott et al., 2011). Cytokines were removed on day 5; at day 7, embryoid bodies were transferred to 96-well flat-bottom adherent tissue culture-treated plates (BD Falcon) containing APEL medium lacking PVA (AEL medium) (Ng et al., 2008). After 30–35 days of differentiation, embryoid bodies were dissociated with TrypLE Select (Invitrogen), stained with the relevant antibodies, and analyzed with FACSDIVA software (BD Biosciences) on either the BD LSRII or BD LSRFortessa cell analyzers (BD Biosciences), fitted with a BD high-throughput sampler (HTS) module.

For thymic endoderm differentiation assays, cells were initially cultured in APEL or BPEL medium (Ng et al., 2008), supplemented with 150 ng ml^{-1} human Activin A. On day 5, Activin A-containing APEL or BPEL medium was replaced with APEL or BPEL medium alone. On day 7, EBs were transferred to 96-well flat-bottom adherent plates (BD Falcon) in AEL or BEL medium (media lacking

PVA) (Ng et al., 2008). AEL or BEL medium supplemented with 40 ng ml^{-1} human keratinocyte growth factor (KGF; R&D Systems) was used to replenish the cultures on days 14, 21, 28, and 35. Embryoid bodies were harvested for analysis by flow cytometry at the times indicated. For experiments using *MIXL1*$^{GFP/w}$ hESCs (Davis et al., 2008), cells were differentiated in BPEL medium supplemented either with 150 ng ml^{-1} Activin A, 20 ng ml^{-1} human BMP4, and 100 ng ml^{-1} Activin A, or with 100 ng ml^{-1} FGF2 (Peprotech). In all instances, hESC and iPSC cultures and differentiations were maintained at 37°C, in a 5% CO_2/air environment.

Flow Cytometric Analysis and Sorting

For analysis and sorting of live cells, hESCs and embryoid bodies were dissociated to a single-cell suspension using TrypLE Select (Invitrogen), filtered through a 35 μM cell-strainer cap (BD Falcon), and labeled with the appropriate antibodies (see the Supplemental Experimental Procedures) as previously described (Davis et al., 2008). Flow cytometry was performed using a FACSCalibur, FACSDiva, or Influx Cell Sorter (all from BD Biosciences). Flow cytometric gates were set using unmodified hESCs or *FOXN1*$^{GFP/w}$-targeted cells labeled with an appropriate isotype control antibody. Live cells were identified on the basis of forward scatter, side scatter, and propidium iodide (PI) exclusion.

Intracellular flow cytometry using mouse anti-human OCT4 (clone C-10; Santa Cruz Biotechnology) and mouse anti-human FOXN1 (clone E-3; Santa Cruz Biotechnology) antibodies was performed essentially as described by Davis et al. (2008) and as detailed in the Supplemental Experimental Procedures.

Culture of Sorted Cell Aggregates

Cells purified by flow cytometry were aggregated by centrifugation for 5 min at 1,500 rpm, using the spin EB protocol (5–10 × 10^3 cells/well) in BPEL medium supplemented with 5 μM Y27632 ROCK inhibitor, as previously described (Goulburn et al., 2011). Aggregated cell clusters were cultured on gelatin-coated wells of a 96-well flat-bottom adherent plate in BEL medium supplemented with 40 ng ml^{-1} KGF.

Quantitative Real-Time PCR and Microarray Gene Expression Analysis

Total RNA was prepared using a High Pure RNA Isolation Kit (Roche), in accordance with the manufacturer's instructions. Quantitative real-time PCR was performed essentially as described by Pick et al. (2007), with probes detailed in the Supplemental Experimental Procedures. For microarray analysis, RNA samples were amplified, labeled, and hybridized to the Human WG-6 (v. 3.0) BeadChip and the Human HT-12 (v. 3.0) BeadChip (Illumina) at the Australian Genome Research Facility (The Walter and Eliza Hall Institute of Medical Research). Data were analyzed using GenomeStudio Gene Expression Module (v. 1.5.4) (Illumina) using average normalization across all samples, with additional analysis performed using GeneSpring GX software (Agilent Technologies), as previously described (Goulburn et al., 2011).

IFN-γ Stimulation Assay

Human IFN-γ (R&D Systems) at 10 ng ml^{-1} was added to day 30 thymic differentiation cultures in BEL medium. After 72 hr of IFN-γ stimulation, differentiated cells were harvested and analyzed for HLA-DR expression by flow cytometry.

$CD34^+CD7^+$ proT-Cell and FOXN1-GFP^+ TEC Progenitor Coculture Assays

Umbilical cord blood (UCB) mononuclear cells were obtained and processed as previously described (La Motte-Mohs et al., 2005). They were pre-enriched into lineage-negative (Lin^-) fractions, sorted into $CD34^+$ hematopoietic stem cells, and cultured on OP9-DL1 cells for 9–10 days, as previously described (Awong et al., 2009). $CD45^+CD34^+CD7^+$ proT-cells were FACS purified and either cultured alone (1 × 10^3 cells/well) or with FOXN1-GFP^+ (3.5 × 10^3 cells/well) or GFP^- (3.5 × 10^3 cells/well) cells in 96-well round-bottom low-attachment plates (Costar), containing APEL medium supplemented with 20% FBS, rhFLT3L (5 ng ml^{-1}, Peprotech), rhIL7 (5 ng ml^{-1}, Peprotech), and rhSCF (30 ng ml^{-1}, Peprotech). A half-media change was performed every 3–4 days. Cultures were analyzed by flow cytometry using the indicated antibodies and as detailed in the Supplemental Experimental Procedures.

ACCESSION NUMBERS

The GEO accession number for the microarray data reported in this paper is GSE56373.

SUPPLEMENTAL INFORMATION

Supplemental Information includes Supplemental Experimental Procedures, five figures, and two tables and can be found with this article online at http://dx.doi.org/10.1016/j.stemcr.2014.04.009.

ACKNOWLEDGMENTS

The authors thank Robyn Mayberry and the staff of StemCore Victoria for the provision of hESCs and iPSCs and Andrew Fryga and the staff of FlowCore (Monash University) for flow cytometric sorting. They also thank Jade Homann and the staff of Monash University Animal Research Laboratories for assistance with mouse experiments and Dr. Mahmood Mohtashami for technical advice on proT-cell cocultures. This work was supported by grants from the Australian Stem Cell Centre, Stem Cells Australia, the Juvenile Diabetes Research Foundation, the National Health and Medical Research Council of Australia (NHMRC), and the Victoria-California Stem Cell Alliance. K.K. is a recipient of an Alexander Graham Bell Doctoral Award from the Natural Sciences and Engineering Research Council of Canada. J.C.Z.-P. is supported by a Canada Research Chair in Developmental Immunology. S.K.N. and D.N.H. are supported by Australian Research Council Future Fellowships. A.G.E. and E.G.S. are Senior Research Fellows of the NHMRC.

Received: November 21, 2013
Revised: April 15, 2014
Accepted: April 16, 2014
Published: May 22, 2014

REFERENCES

Anderson, G., Lane, P.J., and Jenkinson, E.J. (2007). Generating intrathymic microenvironments to establish T-cell tolerance. Nat. Rev. Immunol. *7*, 954–963.

Awong, G., Herer, E., Surh, C.D., Dick, J.E., La Motte-Mohs, R.N., and Zúñiga-Pflücker, J.C. (2009). Characterization in vitro and engraftment potential in vivo of human progenitor T cells generated from hematopoietic stem cells. Blood *114*, 972–982.

Beaudette-Zlatanova, B.C., Knight, K.L., Zhang, S., Stiff, P.J., Zúñiga-Pflücker, J.C., and Le, P.T. (2011). A human thymic epithelial cell culture system for the promotion of lymphopoiesis from hematopoietic stem cells. Exp. Hematol. *39*, 570–579.

Cai, J., Zhao, Y., Liu, Y., Ye, F., Song, Z., Qin, H., Meng, S., Chen, Y., Zhou, R., Song, X., et al. (2007). Directed differentiation of human embryonic stem cells into functional hepatic cells. Hepatology *45*, 1229–1239.

Candi, E., Rufini, A., Terrinoni, A., Giamboi-Miraglia, A., Lena, A.M., Mantovani, R., Knight, R., and Melino, G. (2007). DeltaNp63 regulates thymic development through enhanced expression of FgfR2 and Jag2. Proc. Natl. Acad. Sci. USA *104*, 11999–12004.

Costa, M., Dottori, M., Ng, E., Hawes, S.M., Sourris, K., Jamshidi, P., Pera, M.F., Elefanty, A.G., and Stanley, E.G. (2005). The hESC line Envy expresses high levels of GFP in all differentiated progeny. Nat. Methods *2*, 259–260.

Costa, M., Dottori, M., Sourris, K., Jamshidi, P., Hatzistavrou, T., Davis, R., Azzola, L., Jackson, S., Lim, S.M., Pera, M., et al. (2007). A method for genetic modification of human embryonic stem cells using electroporation. Nat. Protoc. *2*, 792–796.

Costa, M., Sourris, K., Hatzistavrou, T., Elefanty, A.G., and Stanley, E.G. (2008). Expansion of human embryonic stem cells in vitro. Curr. Protoc. Stem Cell Biol. *Chapter 1*, 1C.1.1–1C.1.7.

D'Amour, K.A., Agulnick, A.D., Eliazer, S., Kelly, O.G., Kroon, E., and Baetge, E.E. (2005). Efficient differentiation of human embryonic stem cells to definitive endoderm. Nat. Biotechnol. *23*, 1534–1541.

D'Amour, K.A., Bang, A.G., Eliazer, S., Kelly, O.G., Agulnick, A.D., Smart, N.G., Moorman, M.A., Kroon, E., Carpenter, M.K., and Baetge, E.E. (2006). Production of pancreatic hormone-expressing endocrine cells from human embryonic stem cells. Nat. Biotechnol. *24*, 1392–1401.

Dalton, D.K., Pitts-Meek, S., Keshav, S., Figari, I.S., Bradley, A., and Stewart, T.A. (1993). Multiple defects of immune cell function in mice with disrupted interferon-gamma genes. Science *259*, 1739–1742.

Davis, R.P., Ng, E.S., Costa, M., Mossman, A.K., Sourris, K., Elefanty, A.G., and Stanley, E.G. (2008). Targeting a GFP reporter gene to the MIXL1 locus of human embryonic stem cells identifies human primitive streak-like cells and enables isolation of primitive hematopoietic precursors. Blood *111*, 1876–1884.

Derbinski, J., Gäbler, J., Brors, B., Tierling, S., Jonnakuty, S., Hergenhahn, M., Peltonen, L., Walter, J., and Kyewski, B. (2005). Promiscuous gene expression in thymic epithelial cells is regulated at multiple levels. J. Exp. Med. *202*, 33–45.

Drukker, M., Katz, G., Urbach, A., Schuldiner, M., Markel, G., Itskovitz-Eldor, J., Reubinoff, B., Mandelboim, O., and Benvenisty, N. (2002). Characterization of the expression of MHC proteins in human embryonic stem cells. Proc. Natl. Acad. Sci. USA *99*, 9864–9869.

Elliott, D.A., Braam, S.R., Koutsis, K., Ng, E.S., Jenny, R., Lagerqvist, E.L., Biben, C., Hatzistavrou, T., Hirst, C.E., Yu, Q.C., et al. (2011). NKX2-5(eGFP/w) hESCs for isolation of human cardiac progenitors and cardiomyocytes. Nat. Methods *8*, 1037–1040.

Erickson, M., Morkowski, S., Lehar, S., Gillard, G., Beers, C., Dooley, J., Rubin, J.S., Rudensky, A., and Farr, A.G. (2002). Regulation of thymic epithelium by keratinocyte growth factor. Blood *100*, 3269–3278.

Giancotti, F.G. (1996). Signal transduction by the alpha 6 beta 4 integrin: charting the path between laminin binding and nuclear events. J. Cell Sci. *109*, 1165–1172.

Gordon, J., Bennett, A.R., Blackburn, C.C., and Manley, N.R. (2001). Gcm2 and Foxn1 mark early parathyroid- and thymus-specific domains in the developing third pharyngeal pouch. Mech. Dev. *103*, 141–143.

Goulburn, A.L., Alden, D., Davis, R.P., Micallef, S.J., Ng, E.S., Yu, Q.C., Lim, S.M., Soh, C.L., Elliott, D.A., Hatzistavrou, T., et al. (2011). A targeted NKX2.1 human embryonic stem cell reporter line enables identification of human basal forebrain derivatives. Stem Cells *29*, 462–473.

Gouon-Evans, V., Boussemart, L., Gadue, P., Nierhoff, D., Koehler, C.I., Kubo, A., Shafritz, D.A., and Keller, G. (2006). BMP-4 is required for hepatic specification of mouse embryonic stem cell-derived definitive endoderm. Nat. Biotechnol. *24*, 1402–1411.

Green, M.D., Chen, A., Nostro, M.C., d'Souza, S.L., Schaniel, C., Lemischka, I.R., Gouon-Evans, V., Keller, G., and Snoeck, H.W. (2011). Generation of anterior foregut endoderm from human embryonic and induced pluripotent stem cells. Nat. Biotechnol. *29*, 267–272.

Greenbaum, A., Hsu, Y.M., Day, R.B., Schuettpelz, L.G., Christopher, M.J., Borgerding, J.N., Nagasawa, T., and Link, D.C. (2013). CXCL12 in early mesenchymal progenitors is required for haematopoietic stem-cell maintenance. Nature *495*, 227–230.

Jerome, L.A., and Papaioannou, V.E. (2001). DiGeorge syndrome phenotype in mice mutant for the T-box gene, Tbx1. Nat. Genet. *27*, 286–291.

Kroon, E., Martinson, L.A., Kadoya, K., Bang, A.G., Kelly, O.G., Eliazer, S., Young, H., Richardson, M., Smart, N.G., Cunningham, J., et al. (2008). Pancreatic endoderm derived from human embryonic stem cells generates glucose-responsive insulin-secreting cells in vivo. Nat. Biotechnol. *26*, 443–452.

Kubo, A., Shinozaki, K., Shannon, J.M., Kouskoff, V., Kennedy, M., Woo, S., Fehling, H.J., and Keller, G. (2004). Development of definitive endoderm from embryonic stem cells in culture. Development *131*, 1651–1662.

La Motte-Mohs, R.N., Herer, E., and Zúñiga-Pflücker, J.C. (2005). Induction of T-cell development from human cord blood hematopoietic stem cells by Delta-like 1 in vitro. Blood *105*, 1431–1439.

Lai, L., and Jin, J. (2009). Generation of thymic epithelial cell progenitors by mouse embryonic stem cells. Stem Cells *27*, 3012–3020.

Laster, A.J., Itoh, T., Palker, T.J., and Haynes, B.F. (1986). The human thymic microenvironment: thymic epithelium contains specific keratins associated with early and late stages of epidermal keratinocyte maturation. Differentiation *31*, 67–77.

Liu, C., Ueno, T., Kuse, S., Saito, F., Nitta, T., Piali, L., Nakano, H., Kakiuchi, T., Lipp, M., Hollander, G.A., and Takahama, Y. (2005). The role of CCL21 in recruitment of T-precursor cells to fetal thymi. Blood *105*, 31–39.

Longmire, T.A., Ikonomou, L., Hawkins, F., Christodoulou, C., Cao, Y., Jean, J.C., Kwok, L.W., Mou, H., Rajagopal, J., Shen, S.S., et al. (2012). Efficient derivation of purified lung and thyroid progenitors from embryonic stem cells. Cell Stem Cell *10*, 398–411.

Mainiero, F., Colombara, M., Antonini, V., Strippoli, R., Merola, M., Poffe, O., Tridente, G., and Ramarli, D. (2003). p38 MAPK is a critical regulator of the constitutive and the beta4 integrin-regulated expression of IL-6 in human normal thymic epithelial cells. Eur. J. Immunol. *33*, 3038–3048.

Min, D., Panoskaltsis-Mortari, A., Kuro-O, M., Holländer, G.A., Blazar, B.R., and Weinberg, K.I. (2007). Sustained thymopoiesis and improvement in functional immunity induced by exogenous KGF administration in murine models of aging. Blood *109*, 2529–2537.

Mohtashami, M., Shah, D.K., Nakase, H., Kianizad, K., Petrie, H.T., and Zúñiga-Pflücker, J.C. (2010). Direct comparison of Dll1- and Dll4-mediated Notch activation levels shows differential lymphomyeloid lineage commitment outcomes. J. Immunol. *185*, 867–876.

Nehls, M., Pfeifer, D., Schorpp, M., Hedrich, H., and Boehm, T. (1994). New member of the winged-helix protein family disrupted in mouse and rat nude mutations. Nature *372*, 103–107.

Nehls, M., Kyewski, B., Messerle, M., Waldschütz, R., Schüddekopf, K., Smith, A.J., and Boehm, T. (1996). Two genetically separable steps in the differentiation of thymic epithelium. Science *272*, 886–889.

Ng, E.S., Davis, R., Stanley, E.G., and Elefanty, A.G. (2008). A protocol describing the use of a recombinant protein-based, animal product-free medium (APEL) for human embryonic stem cell differentiation as spin embryoid bodies. Nat. Protoc. *3*, 768–776.

Parent, A.V., Russ, H.A., Khan, I.S., LaFlam, T.N., Metzger, T.C., Anderson, M.S., and Hebrok, M. (2013). Generation of functional thymic epithelium from human embryonic stem cells that supports host T cell development. Cell Stem Cell *13*, 219–229.

Pick, M., Azzola, L., Mossman, A., Stanley, E.G., and Elefanty, A.G (2007). Differentiation of human embryonic stem cells in serum-free medium reveals distinct roles for bone morphogenetic protein 4, vascular endothelial growth factor, stem cell factor, and fibroblast growth factor 2 in hematopoiesis. Stem Cells *25*, 2206–2214.

Pignata, C., Fiore, M., Guzzetta, V., Castaldo, A., Sebastio, G., Porta, F., and Guarino, A. (1996). Congenital Alopecia and nail dystrophy associated with severe functional T-cell immunodeficiency in two sibs. Am. J. Med. Genet. *65*, 167–170.

Richards, M., Fong, C.Y., Chan, W.K., Wong, P.C., and Bongso, A. (2002). Human feeders support prolonged undifferentiated growth of human inner cell masses and embryonic stem cells. Nat. Biotechnol. *20*, 933–936.

Rossi, S.W., Jeker, L.T., Ueno, T., Kuse, S., Keller, M.P., Zuklys, S., Gudkov, A.V., Takahama, Y., Krenger, W., Blazar, B.R., and Holländer, G.A. (2007). Keratinocyte growth factor (KGF) enhances postnatal T-cell development via enhancements in proliferation and function of thymic epithelial cells. Blood *109*, 3803–3811.

Shezen, E., Okon, E., Ben-Hur, H., and Abramsky, O. (1995). Cytokeratin expression in human thymus: immunohistochemical mapping. Cell Tissue Res. *279*, 221–231.

Sun, X., Xu, J., Lu, H., Liu, W., Miao, Z., Sui, X., Liu, H., Su, L., Du, W., He, Q., et al. (2013). Directed differentiation of human embryonic stem cells into thymic epithelial progenitor-like cells reconstitutes the thymic microenvironment in vivo. Cell Stem Cell *13*, 230–236.

Tada, S., Era, T., Furusawa, C., Sakurai, H., Nishikawa, S., Kinoshita, M., Nakao, K., Chiba, T., and Nishikawa, S. (2005). Characterization of mesendoderm: a diverging point of the definitive endoderm and mesoderm in embryonic stem cell differentiation culture. Development *132*, 4363–4374.

Thomson, J.A., Itskovitz-Eldor, J., Shapiro, S.S., Waknitz, M.A., Swiergiel, J.J., Marshall, V.S., and Jones, J.M. (1998). Embryonic stem cell lines derived from human blastocysts. Science *282*, 1145–1147.

Walsh, E.C., Mather, K.A., Schaffner, S.F., Farwell, L., Daly, M.J., Patterson, N., Cullen, M., Carrington, M., Bugawan, T.L., Erlich, H., et al. (2003). An integrated haplotype map of the human major histocompatibility complex. Am. J. Hum. Genet. *73*, 580–590.

Yang, S.J., Ahn, S., Park, C.S., Holmes, K.L., Westrup, J., Chang, C.H., and Kim, M.G. (2006). The quantitative assessment of MHC II on thymic epithelium: implications in cortical thymocyte development. Int. Immunol. *18*, 729–739.

Yasunaga, M., Tada, S., Torikai-Nishikawa, S., Nakano, Y., Okada, M., Jakt, L.M., Nishikawa, S., Chiba, T., Era, T., and Nishikawa, S. (2005). Induction and monitoring of definitive and visceral endoderm differentiation of mouse ES cells. Nat. Biotechnol. *23*, 1542–1550.

Yu, J., Hu, K., Smuga-Otto, K., Tian, S., Stewart, R., Slukvin, I.I., and Thomson, J.A. (2009). Human induced pluripotent stem cells free of vector and transgene sequences. Science *324*, 797–801.

Stem Cell Reports, Volume *2*

Supplemental Information

FOXN1$^{GFP/w}$ Reporter hESCs Enable Identification of Integrin-β4, HLA-DR, and EpCAM as Markers of Human PSC-Derived FOXN1^{+} Thymic Epithelial Progenitors

Chew-Li Soh, Antonietta Giudice, Robert A. Jenny, David A. Elliott, Tanya Hatzistavrou, Suzanne J. Micallef, Korosh Kianizad, Natalie Seach, Juan Carlos Zúñiga-Pflücker, Ann P. Chidgey, Alan Trounson, Susan K. Nilsson, David N. Haylock, Richard L. Boyd, Andrew G. Elefanty, and Edouard G. Stanley

Supplemental Experimental Procedures

FOXN1 **targeting vector construction:** The *FOXN1* targeting vector was assembled using standard cloning techniques and Red/ET Recombination technology (Gene Bridges). DNA fragments representing the 5' and 3' homology arms were derived from bacterial artificial chromosome (BAC) RP11-915B21 spanning the human *FOXN1* genomic locus (Roswell Park Cancer Institute). *AscI* and *ClaI* restriction endonuclease sites were introduced during recombineering steps to facilitate subcloning of a *GFP-PGK-neo*^{R} reporter/selection cassette into a pBR322-based targeting vector backbone, while *SwaI* restriction sites were introduced to allow linearization of the construct.

Generation of FOXN1$^{GFP/w}$ hESC Reporter lines: 10^7 HES3 (Richards et al., 2002) or MEL1 (Millipore catalogue no. SCC020) hESCs were electroporated with 25μg of the *FOXN1* targeting vector and subjected to G418 (Geneticin, Gibco) selection. Resultant G418 resistant colonies were screened for homologous recombination events using a PCR-based strategy incorporating the forward primer *Neo4* (5'-cgatgcctgcttgccgaatatc-3') and the reverse primer *FOXN1 26063r* (5'-gcttgactttgacctctgaacc-3') (*primer pair b*, **Figures 1A and 1B**). Six (of 134) MEL1 and three (of 144) HES3 correctly targeted clones were obtained. Homologous integration of the 5' homology arm was confirmed by PCR amplification using the forward primer *FOXN1 10546f* (5'-cattcccagaccccgagtttccttg-3') and the reverse primer *GFP Reverse2* (5'-ccggtgaacagctcctcgcccttgc-3') (*primer pair a*, **Figures 1A and 1B; Figure S1A**). The loxP-flanked *neo*^{R} cassette was removed by Cre recombinase-mediated excision as previously described (Davis et al., 2008). Sub-clones that had excised the *neo*^{R} gene were identified using the PCR primer *GFP1* (5'-gtgctgctgcccgacaaccactac-3') in combination with the reverse primer *FOXN1 26063r*, which generated a product that spanned the site formally occupied by the *neo*^{R} cassette (*primer pair c*, **Figures 1A and 1B; Figure S1A**). Correctly targeted clones that had excised the selectable marker were single cell cloned as previously described (Davis et al., 2008).

Antibodies used in this study: Primary antibodies used were mouse anti-human E-CADHERIN (Invitrogen), mouse anti-human SSEA-4 (Chemicon International), mouse anti-human TRA-1-60 (Chemicon International), mouse anti-human PDGFRα (BD Pharmingen), mouse anti-human CD104 (BD Pharmingen), fluorescein isothiocyanate (FITC)-conjugated mouse anti-human CD9 (BD Pharmingen), phycoerythrin (PE)-conjugated mouse anti-human EpCAM (BD Biosciences), and allophycocyanin (APC)-conjugated mouse anti-human HLA-DR (BD Pharmingen). Unconjugated primary antibodies were detected with either APC- or PE-conjugated goat anti-mouse IgG (BD Biosciences).

Intracellular Flow Cytometry: Following dissociation and filtration, cells were fixed and permeabilized with BD Cytofix/Cytoperm (BD Biosciences) at 4°C for 20 min. Cells were washed and stained with primary antibody or an isotype control diluted in 1X BD Perm/Wash (BD Biosciences) at 4°C for 30 min. Unconjugated OCT4 and FOXN1 primary antibodies were detected with APC-conjugated goat anti-mouse IgG (BD Pharmingen). Cells were incubated with secondary antibodies at 4°C for 30 min in the dark. Antibody staining was quantified using the BD FACSCalibur. Forward and side scatter were used to identify cells that were viable immediately prior to the initial fixation process. Flow cytometric analysis was performed using Flowlogic Software (Inivai Technologies).

Quantitative Real-Time Polymerase Chain Reaction (q-PCR): Total RNA was prepared using High Pure RNA Isolation Kit (Roche), according to manufacturer's instructions. First-strand cDNA was reverse-transcribed from RNA samples with random hexamer priming using SuperScript III reagents (Invitrogen), as previously described (Pick et al., 2007). Q-PCR was performed using TaqMan Gene Expression Assays with TaqMan Universal PCR Master Mix (Applied Biosystems) on the 7500 Fast Real Time PCR System (Applied Biosystems) under standard cycling parameters according to manufacturer's instructions. The TaqMan Gene Expression Assays employed in this study were *FOXN1* (Hs00186096_m1), *GAPDH* (Hs99999905_m1), *FOXA2* (Hs00232764_m1), *SOX17* (Hs00751752_s1), *HOXA3* (Hs00601076_m1), *PAX9* (Hs Hs00196354_m1), *FGFR2* (Hs01552926_m1), *INVOLUCRIN* (Hs00902520_m1), *KERATIN-10* (Hs00166289_m1), *KERATIN-5* (Hs00361185_m1), *KERATIN-8* (Hs01670053_m1) and *GCM2* (Hs00171702_m1). The comparative cycle threshold (Ct) method was used to analyze data, with gene expression levels normalized against input determined by reference to the *GAPDH* housekeeping gene, with an arbitrary multiplier of 10,000 for sample normalization, as previously published (Pick et al., 2007).

FOXN1-GFP$^+$ TEC progenitor - CD34$^+$CD7$^+$ proT-cell co-culture assays: Sorted CD34$^+$ cells from Lin$^-$ UCB were cultured on OP9-DL1 cells for 9-10 days in OP9 Medium containing rhFLT3L (5 ng ml^{-1}, Peprotech), rhIL7 (5 ng ml^{-1}, Peprotech) and rhSCF (30 ng ml^{-1}, Peprotech) (Awong et al., 2009; La Motte-Mohs et al., 2005). FACS purified CD45$^+$CD34$^+$CD7$^+$ proT-cells were either cultured alone (1 x 10^3 cells/well) or with FOXN1-GFP$^+$ (3.5 x 10^3 cells/well) or GFP$^-$ (3.5 x 10^3 cells/well) cells in 96-well round-bottom low attachment plates (Costar) containing APEL Medium supplemented with 20% FBS, rhFLT3L (5 ng ml^{-1}), rhIL7 (5 ng ml^{-1}) and rhSCF (30 ng ml^{-1}). A half-media change was performed every 3-4 days. Differentiated proT-cells were analyzed for the expression of CD34, CD1a, CD5, CD4, CD8, CD3, CD45 and CD14 cell surface markers at weekly intervals. The following antibodies (all supplied by BD Pharmingen) were employed for analyses: APC-Cy7- and APC-conjugated mouse anti-human CD45, PE-Cy7- and APC-conjugated mouse anti-human CD34, PE-conjugated mouse anti-human CD7, PE-conjugated mouse anti-human CD1a, Biotin-conjugated mouse anti-human CD5, APC-conjugated mouse anti-human CD4, APC- and PE-conjugated mouse anti-human CD8, APC-conjugated mouse anti-human CD3 and PE-conjugated mouse anti-human CD14. The biotin-conjugated CD5 was detected with a Streptavidin-PE-conjugated secondary antibody.

References for Supplemental Material

Awong G., Herer E., Surh C.D., Dick J.E., La Motte-Mohs R.N., and Zuniga-Pflucker J.C. (2009). Characterization in vitro and engraftment potential in vivo of human progenitor T cells generated from hematopoietic stem cells. Blood *114*, 972-982.

Davis R.P., Ng E.S., Costa M., Mossman A.K., Sourris K., Elefanty A.G., and Stanley E.G. (2008). Targeting a GFP reporter gene to the MIXL1 locus of human embryonic stem cells identifies human primitive streak-like cells and enables isolation of primitive hematopoietic precursors. Blood *111*, 1876-1884.

La Motte-Mohs R.N., Herer E., and Zuniga-Pflucker J.C. (2005). Induction of T-cell development from human cord blood hematopoietic stem cells by Delta-like 1 in vitro. Blood *105*, 1431-1439.

Pick M., Azzola L., Mossman A., Stanley E.G., and Elefanty A.G. (2007). Differentiation of human embryonic stem cells in serum-free medium reveals distinct roles for bone morphogenetic protein 4, vascular endothelial growth factor, stem cell factor, and fibroblast growth factor 2 in hematopoiesis. Stem Cells *25*, 2206-2214.

Richards M., Fong C.Y., Chan W.K., Wong P.C., and Bongso A. (2002). Human feeders support prolonged undifferentiated growth of human inner cell masses and embryonic stem cells. Nat. Biotechnol. *20*, 933-936.
